# DNA damage response induced by Etoposide promotes steroidogenesis via GADD45A in cultured adrenal cells

**DOI:** 10.1038/s41598-018-27938-5

**Published:** 2018-06-25

**Authors:** Mimi Tamamori-Adachi, Akane Koga, Takao Susa, Hiroko Fujii, Masao Tsuchiya, Hiroko Okinaga, Harumi Hisaki, Masayoshi Iizuka, Shigetaka Kitajima, Tomoki Okazaki

**Affiliations:** 1 Department of Biochemistry, Teikyo University School of Medicine, 2-11-1, Kaga, Itabashi-ku, Tokyo 173-8605 Japan; 2 Department of Practical Pharmacy, Faculty of Pharmaceutical Sciences, Teikyo University, 2-11-1, Kaga, Itabashi-ku, Tokyo 173-8605 Japan; 3 Department of General Medicine, National Defense Medical College, 3-2, Namiki, Tokorozawa City, Saitama 359-8513 Japan; 4 Department of Internal Medicine, Teikyo University School of Medicine, 2-11-1, Kaga, Itabashi-ku, Tokyo 173-8605 Japan; 5 Department of Biochemical Genetics, Medical Research Institute, Tokyo Medical and Dental University, 1-5-45, Yushima, Bunkyo-ku, Tokyo 113-8605 Japan

## Abstract

Glucocorticoid production is regulated by adrenocorticotropic hormone (ACTH) via the cyclic adenosine monophosphate (cAMP)/protein kinase A (PKA) pathway in the adrenal cortex, but the changes in steroidogenesis associated with aging are unknown. In this study, we show that cell-autonomous steroidogenesis is induced by non-ACTH- mediated genotoxic stress in human adrenocortical H295R cells. Low-dose etoposide (EP) was used to induce DNA damage as a genotoxic stress, leading to cellular senescence. We found that steroidogenesis was promoted in cells stained with γH2AX, a marker of DNA damaged cells. Among stress-associated and p53-inducible genes, the expression of GADD45A and steroidogenesis-related genes was significantly upregulated. Immunofluorescence analysis revealed that GADD45A accumulated in the nuclei. Metabolite assay using cultured media showed that EP-treated cells were induced to produce and secrete considerable amounts of glucocorticoid. Knockdown of GADD45A using small interfering RNA markedly inhibited the EP-induced upregulation of steroidogenesis-related gene expression, and glucocorticoid production. A p38MAPK inhibitor, but not a PKA inhibitor, suppressed EP-stimulated steroidogenesis. These results suggest that DNA damage itself promotes steroidogenesis via one or more unprecedented non-ACTH-mediated pathway. Specifically, GADD45A plays a crucial role in the steroidogenic processes triggered by EP-stimulated genotoxic stress. Our study sheds new light on an alternate mechanism of steroidogenesis in the adrenal cortex.

## Introduction

Steroid hormones are synthesized in steroidogenic cells of the adrenal gland, ovary, testis, placenta, and brain and are required for normal reproductive function and various branches of metabolic and physiological homeostasis. Steroid biosynthesis is fine-tuned by the phosphorylation-dephosphorylation cycles of various intermediate proteins. In these processes, phosphorylation-dependent events are required for the acute stimulation of steroid production through the activation of protein kinases, including cyclic adenosine monophosphate (cAMP)-dependent protein kinase A (PKA), protein kinase C (PKC), calcium/calmodulin-dependent protein kinase, and mitogen-activated protein kinases (MAPKs). Then, the subsequent dephosphorylation of each event ensures to make closed loops in order to maintain steroid production within a narrow range for cellular homeostasis^1–6^.

Glucocorticoids are steroid hormones with important functions in the regulation of metabolism, development, and immune responses^7,8^. In particular, their anti-inflammatory properties underpin the concept that glucocorticoid synthesis must be readily turned on and off because the production of too little glucocorticoid may result in the overactivation of immune cells, chronic inflammation, and immunopathology, whereas too much glucocorticoid synthesis may render the host immunosuppressed and thus incapable of responding to pathogens.

Adrenal gland is a key component of the hypothalamus-pituitary-adrenal (HPA) axis, thus playing a crucial role in the adaptation of organisms to a range of different stressors. Through binding to its receptor melanocortin 2 receptor (MC2R), which is located in the adrenal cortical fasciculate layer, adrenocorticotropic hormone (ACTH), another core player of the HPA axis, predominantly activates adenylyl cyclase and leads to cAMP production, followed by PKA activation. Then, subsequent phosphorylation of specific transcription factors activates steroidogenic enzyme expression through an increase in the availability of free cholesterol, steroidogenic acute regulatory protein (StAR), cytochrome P450c11 (encoded by CYP11A1), cytochrome P450c21A2 (encoded by CYP21A2), cytochrome P450c17 (encoded by CYP17A1), and 3β-hydroxysteroid dehydrogenase II (encoded by HSD3B2)^9–17^.

Aged organs are exposed to various stresses such as DNA damage caused by environmental insults including UV irradiation, exogenous chemicals, and biological genotoxins, as well as endogenous sources over a long period of time, resulting in the accumulation of senescent cells^18–21^. Although it is well known that the functions of glucocorticoid are critical to the maintenance of cellular homeostasis, the change of glucocorticoid production in the aged adrenal cortex is less well understood. It has been reported that concentration of glucocorticoid in the serum or salivary is increased in aged mice and human^22–26^. However, the underlying mechanism(s) remains elusive; for example, it may include cellular senescence induced by DNA damage, telomere shortening, oxidative stress, and oncogenes.

To combat DNA damage and maintain cellular homeostasis, cells are equipped with a DNA repair network referred to as the DNA damage response (DDR). As a result, various repair machinery proteins are activated after cell cycle checkpoints^27^. γ-H2AX (i.e., phosphorylated H2AX), which is a variant of histone H2A, represents the presence of DNA double strand breaks (DSBs), irrespective of their origin^24,25^. Thus, γ-H2AX foci are used as surrogates for DNA damage and the scoring of γ-H2AX foci is widely used as a measure for DSBs^28,29^.

One central signaling pathway triggered by the DDR is the activation of the p53 tumor suppressor, leading to cell cycle arrest and apoptosis. Growth arrest and DNA-damaging-induced 45A (GADD45A) is a target of p53 as well as the cyclin-dependent kinase (CDK) inhibitor p21. GADD45A plays an important role in the integration of cellular responses to a wide variety of stressors in mammals^30–34^, and is induced both with and without the help of p53^35,36^. In basal conditions, GADD45A is expressed at a relatively low level, but it is highly inducible by a plethora of stressful stimuli, both physiological and environmental, such as genotoxic and oxidative stress. GADD45A controls the stress response by interacting directly with other proteins to modify their function. Depending on the specific context (cell type, stress, etc.), GADD45A may bind to and regulate certain protein kinases, nucleotide excision repair proteins, base excision repair proteins, nuclear hormone receptors, and/or cell cycle regulators^34,37,38^. Several studies reported that GADD45A is involved in cellular senescence by diverse factors^39–42^. Mainly, by its interaction with and activation of p38MAPK, GADD45A induces p21 expression, resulting in cell cycle arrest and cellular senescence^40–42^.

p38MAPK is activated in response to environmental stress, cytokines, and DNA damage^43–50^. p38MAPK is activated following phosphorylation at Thr^180^ and Tyr^182^ in a Thr-Gly-Tyr motif. This phosphorylation is mediated by the upstream MAPKs (MAPKKs) MKK3 and MKK6, which are in turn activated by several different and overlapping sets of MAPKKKs or MEKKs, including MEKK4, which is activated by binding to GADD45A^51–55^. Thus GADD45A may well activate p38MAPK by interacting with MEKK4, one of the upstream MAPKs of p38MAPK.

In this study, we treated cultured human adrenocortical tumor cells (H295R cells) with a low dose of etoposide (EP) to induce DNA damage as a genotoxic stress, mimicking cellular senescence. H295R cells are transformed human adrenal cortical cells that secrete all of the steroid intermediates of the steroidogenesis pathway^56,57^. We found that steroidogenesis was promoted in an adrenocortical cell-autonomous manner and independently of the HPA axis after the DDR through the activation of GADD45A and p38MAPK pathways. We argue the significance of the mechanism of cell-autonomous steroid production in these cells by the DDR.

## Results

### Induction of steroidogenesis by EP in H295R cells

**Figure 1:**
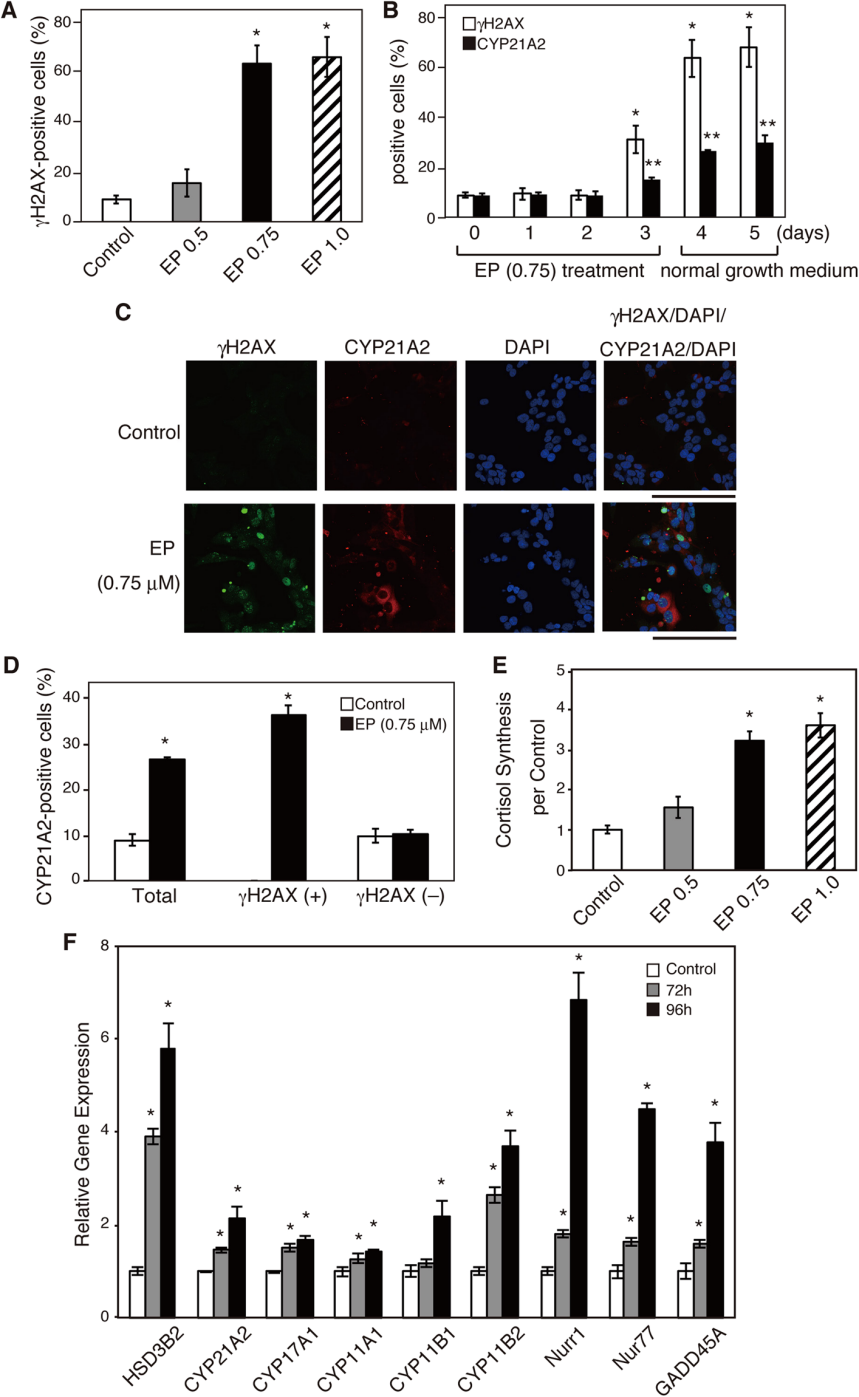
Effects of EP on the induction of DNA damage, CYP21A2 accumulation, cortisol production, and the expression of steroidogenesis-related genes. H295R cells were treated with the indicated concentration of EP (μM) for 72 h (3 days), changed to the normal growth medium, and cultured for another 24–48 h (4 and 5 days). (**A**–**D**) H295R cells were fixed with 4% paraformaldehyde, followed by immunofluorescence. Quantitative analysis (**A**,**B** and **D**) obtained from confocal microscope experiments and images (**C**) are shown. At least 200 cells from several random fields were scored in a blinded manner for each condition. Data are presented as the mean ± standard error (SE) of 3 independent experiments. *P < 0.05 vs. control. (**A**) Quantitative analysis of γH2AX-positive cells 4 days after EP (μM) treatment. (**B**) Quantitative analysis of γH2AX- and CYP21A2- positive cells. (**C**) Immunostaining for γH2AX and CYP21A2 4 days after EP (0.75 μM) treatment. Green staining with the anti-γH2AX antibody, red staining with the anti-CYP21A2 antibody, and blue staining with DAPI (nuclei) are shown. Scale bars represent 100 μm. (**D**) Percentages of CYP21A2-positive cells 4 days after EP (0.75 μM) treatment in total, γH2AX-positive, or -negative cells. (**E**) Cortisol concentration in the medium of the cells treated with EP (μM) 4 days after EP (0.75 μM) treatment was measured using an enzyme-linked immunosorbent assay (ELISA). Data are presented as the mean ± SE of 3 independent experiments. *P < 0.05 vs. control. (**F**) Relative mRNA expression of the indicated genes was analyzed by qRT-PCR. RNA was extracted from H295R cells 3 days (72 h) or 4 days (96 h) after EP (0.75 μM) treatment. Data are presented as the mean ± SE of 3 independent experiments. *P < 0.05 vs. control.

To determine whether the DDR induces steroidogenesis, we treated H295R cells with different concentrations of etoposide (EP) for 3 days (72 h), followed by culturing with normal growth medium. By treatment of 0.75 and 1.0 μM EP, the number of γH2AX-positive cells was significantly increased 4 days after treatment (Fig. 1A and C). Until 2 days after EP treatment, the rate of γH2AX foci-positive cells remained to be approximately 10%. Three days after treatment, the number of γH2AX foci-positive cells began to increase and peaked after 4–5 days (Fig. 1B). Because γH2AX staining is established as a reliable quantitative indicator of the DDR, it was confirmed that DNA damage occurred in the cells 3 days after EP treatment. Interestingly, cells positive for the steroidogenic enzyme CYP21A2 also increased in the same manner as γH2AX foci-positive cells (Fig. 1B and C). As shown in quantitative analyses of double staining of γH2AX and CYP21A2, a remarkable increase in the number of CYP21A2-positive cells by EP treatment was observed in γH2AX-positive cells, but not in γH2AX-negative cells (Fig. 1C and D, and Supplementary Table S1). These findings indicate the strong relationship between EP-induced DDR and the induction of CYP21A2 in H295R cells.

We measured cortisol concentration in the medium of the cells to ensure cortisol production in response to treatment with EP. Cortisol synthesis increased more than 3-times 4 days after treatment (Fig. 1E).

Quantitative real-time PCR (RT-qPCR) experiments were performed using H295R cells treated with or without EP. The levels of HSD3β2, CYP21A2, CYP17A1, CYP11A1, CYP11B1, CYP11B2, Nurr1 and Nur77 mRNA transcript were significantly increased by EP in a time- and dose-dependent manner (Fig. 1F and Supplementary Table S2 and Supplementary Fig. S1). Their expression was not increased until 2 days after the addition of EP. Conversely, the expression of steroidogenic factor 1 (SF1), which plays a major role in regulating steroidogenic enzymes^58^, and StAR was not changed (Supplementary Fig. S2). These data suggest that the DDR induced by EP promotes steroidogenesis in H295R cells and it is accompanied by the upregulation of certain steroidogenesis-related factors.

### Involvement of GADD45A in steroidogenesis in H295R cells

**Figure 2:**
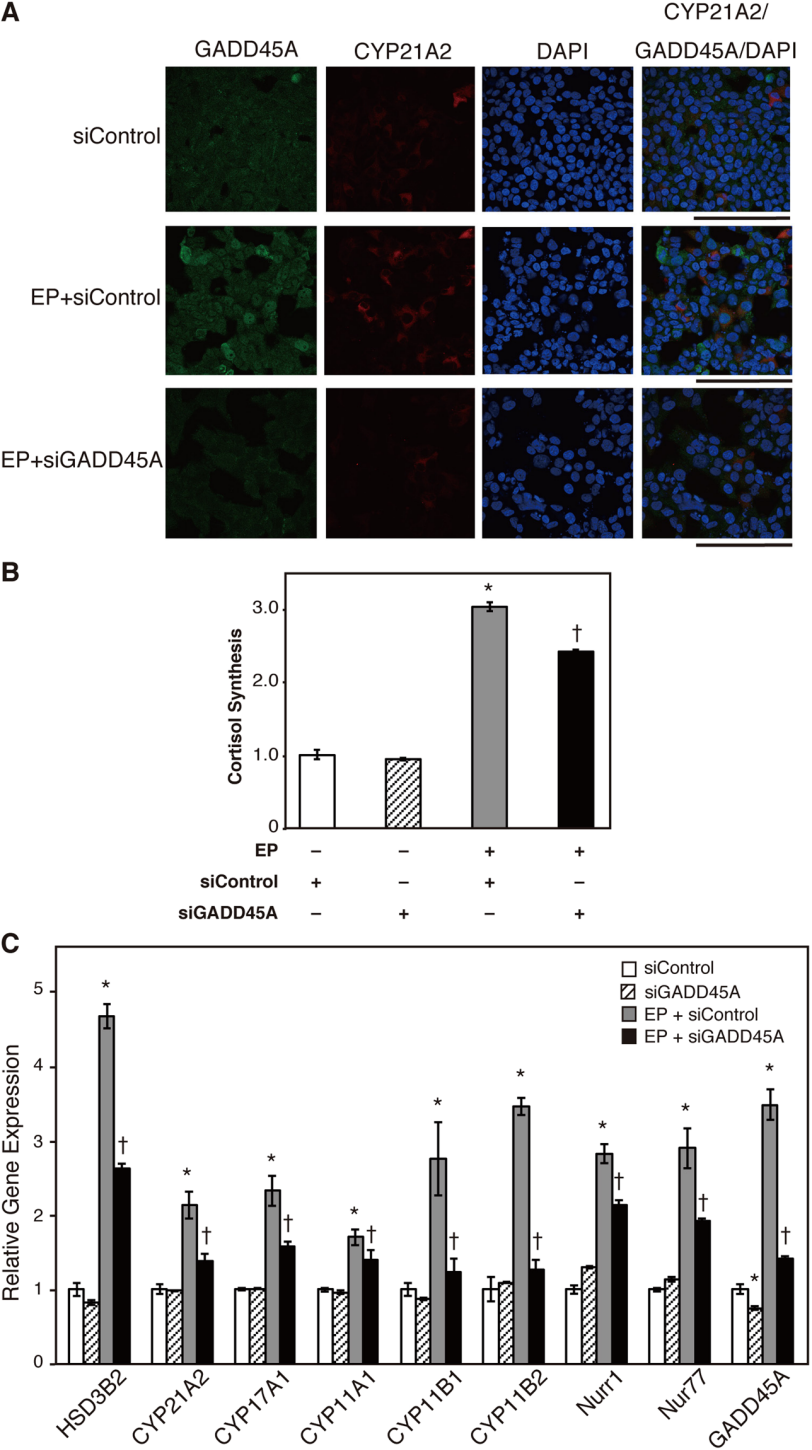
Inhibitory effect of GADD45A siRNA on steroidogenesis promoted by EP. H295R cells were transfected with the indicated siRNAs simultaneously with plating. After 24 h, the cells were treated with EP (0.75 μM) for 72 h, changed to the normal growth medium, and cultured for another 24 h. (**A**) Immunostaining for GADD45A and CYP21A2. H295R cells were fixed with 4% paraformaldehyde. Green staining shows the anti-GADD45A antibody, red staining shows the anti-CYP21A2 antibody, and blue staining shows DAPI (cell nuclei). Scale bars represent 100 μm. (**B**) Cortisol concentration in the medium was measured using ELISA, and corrected by the number of cells. Data are presented as the mean ± SE of 3 independent experiments. *P < 0.05 vs. control, ^†^P < 0.05 vs. EP. (**C**) Relative mRNA expression of the indicated genes was analyzed by RT-qPCR. RNA was extracted at 12 h after changing of the medium (at 84 h after EP-treatment). Data are presented as the mean ± SE of 3 independent experiments. *P < 0.05 vs. control, ^†^P < 0.05 vs. EP + siControl.

**Figure 3:**
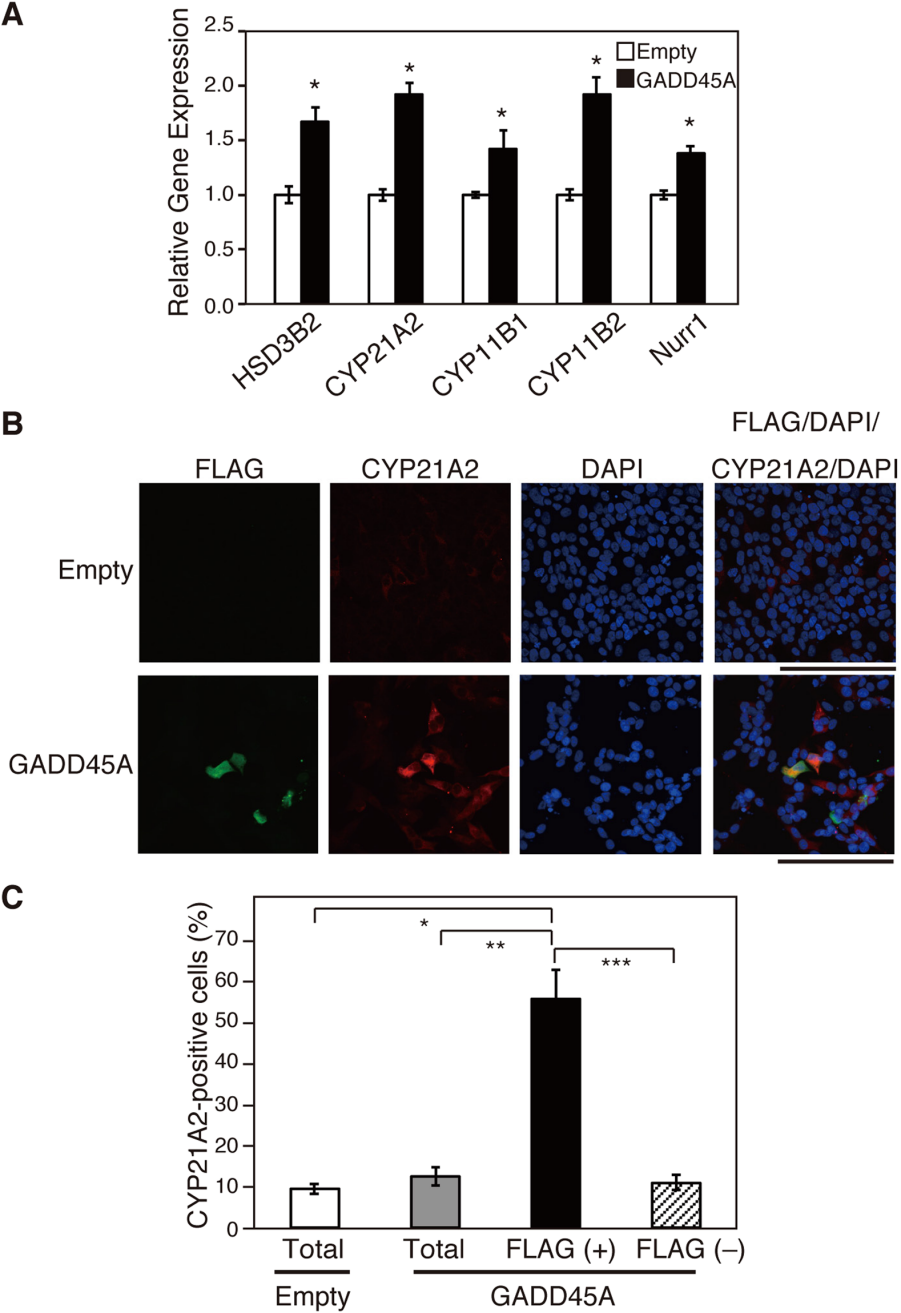
Effect of GADD45A expression on steroidogenesis. H295R cells were transiently transfected with the empty vector or human GADD45A expression vector, and cultured for another 48 h. (**A**) Relative mRNA expression of the indicated genes was analyzed by qRT-PCR. Data are presented as the mean ± SE of 3 independent experiments. *P < 0.05 vs. empty. (**B**) Immunostaining for FLAG and CYP21A2. Green staining shows the anti-FLAG antibody, red staining shows the anti-CYP21A2 antibody, and blue staining shows DAPI (nuclei). Scale bars represent 100 μm. (**C**) The percentages of steroidogenic CYP21A2-positive cells in empty vector-transfected cells (Empty), total FLAG-GADD45A transfected cells (Total), FLAG-GADD45A transfected FLAG-positive [FLAG (+)] or FLAG-GADD45A transfected FLAG-negative [FLAG (−)] cells were measured. At least 200 cells from random fields were examined in each condition in a blinded manner. Data are presented as the mean ± SE of 3 independent experiments. *P < 0.05 vs. Empty, **P < 0.05 vs. Total, ***P < 0.05 vs. FLAG (−).

Having obtained evidence that EP induced steroidogenesis as well as DNA damage in H295R cells, we focused on GADD45A, whose mRNA expression is known to be increased in stressful growth arrest conditions and/or treatment with DNA-damaging agents^30–34^. Our RT-qPCR showed that GADD45A mRNA expression was elevated by 1.61-fold in 72 h EP-treated cells and 3.79-fold in 96 h EP-treated cells (Fig. 1F, and Supplementary Table S2). Further, GADD45A accumulated in the nuclei of approximately 30% of cells treated with EP, whereas control cells did not express GADD45A (Fig. 2A). To demonstrate that GADD45A is involved in EP-induced steroidogenesis, we introduced small interfering RNA (siRNA) against GADD45A (siGADD45A) into H295R cells. As shown in Fig. 2A, siGADD45A decreased not only GADD45A expression but also CYP21A2 expression in EP-treated H295R cells. Moreover, cortisol synthesis was decreased by approximately 20% in the presence of siGADD45A (Figs 2B and S3A). The levels of HSD3B2, CYP21A2, CYP17A1, CYP11A1, CYP11B1, CYP11B2, Nurr1, and Nur77 mRNA transcripts were significantly decreased by siGADD45A (Fig. 2C, Supplementary Table S3, and Supplementary Fig. S3B). Further, we performed transient introduction of a FLAG-tagged GADD45A expression vector into H295R cells and assayed steroidogenesis. As shown in Fig. 3A, HSD3B2, CYP21A2, CYP11B1, CYP11B2, and Nurr1 gene expression was significantly upregulated by approximately 1.7-, 1.9-, 1.4–1.9-, and 1.4-fold, respectively, in GADD45A-transfected H295R cells, in which a greater than 1000-fold increase in GADD45A was confirmed (Fig. 3A and Supplementary Table S4). Immunofluorescence analysis also showed that CYP21A2 expression was induced in those cells (Fig. 3B). Approximately 60% of FLAG-positive cells expressed CYP21A2, whereas it was expressed in around 10% of empty vector-transfected, total FLAG-GADD45A-transfected, or FLAG-GADD45A-transfected FLAG (−) cells (Fig. 3C and Supplementary Table S5), suggesting that CYP21A2 expression is induced predominantly in FLAG-GADD45A-expressing cells. These data suggest that GADD45A is involved in EP-induced steroidogenesis.

### Inhibition of EP-induced steroidogenesis by SB203580 in H295R cells

**Figure 4:**
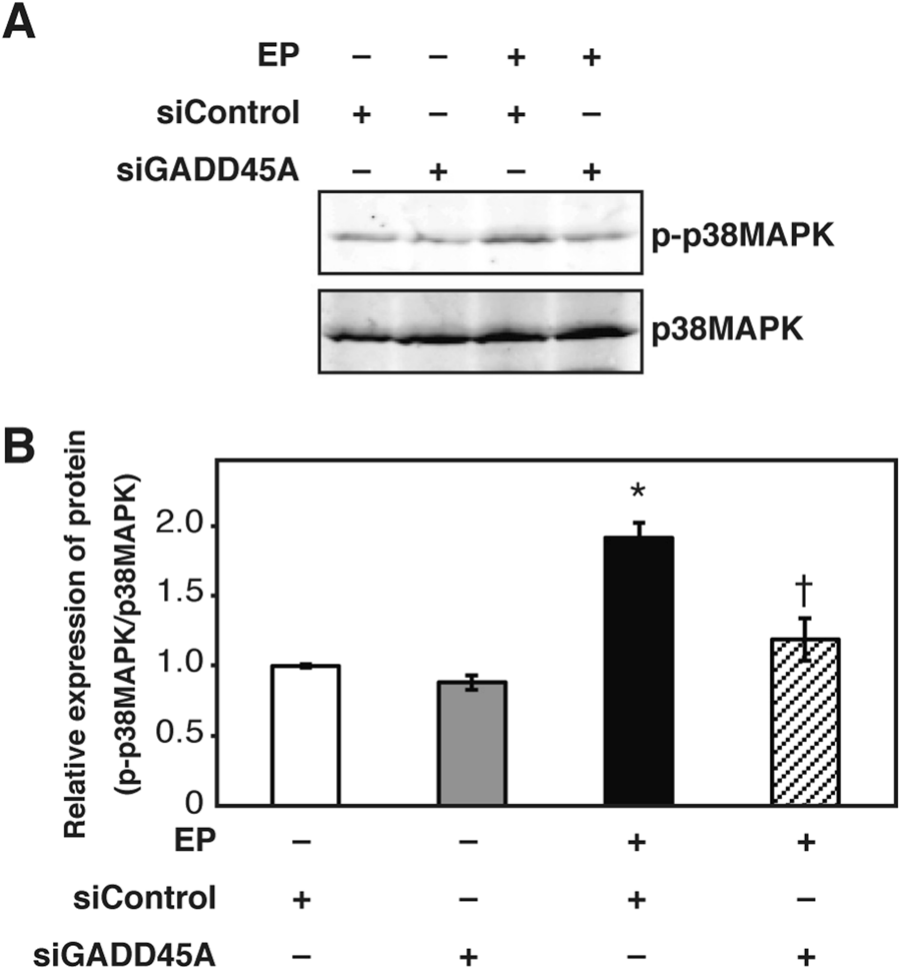
Inhibitory effect of GADD45A siRNA on p38MAPK phosphorylation. H295R cells were transfected with the indicated siRNAs. After 24 h, the cells were treated with EP (0.75 μM) for 72 h, changed to the normal growth medium, and cultured for another 24 h. (**A**) Western blot analysis of phosphorylated p38MAPK (p-p38MAPK) and total p38MAPK (p38MAPK). Cropped images are shown. Uncropped images are shown in Supplementary Fig. S9. (**B**) The level of phosphorylated p38MAPK was normalized by p38MAPK expression. Quantification of the expression level was performed by ImageJ software. Data are presented as the mean ± SE of 3 independent experiments. *P < 0.05 vs. control. ^†^P < 0.05 vs. EP + siControl.

**Figure 5:**
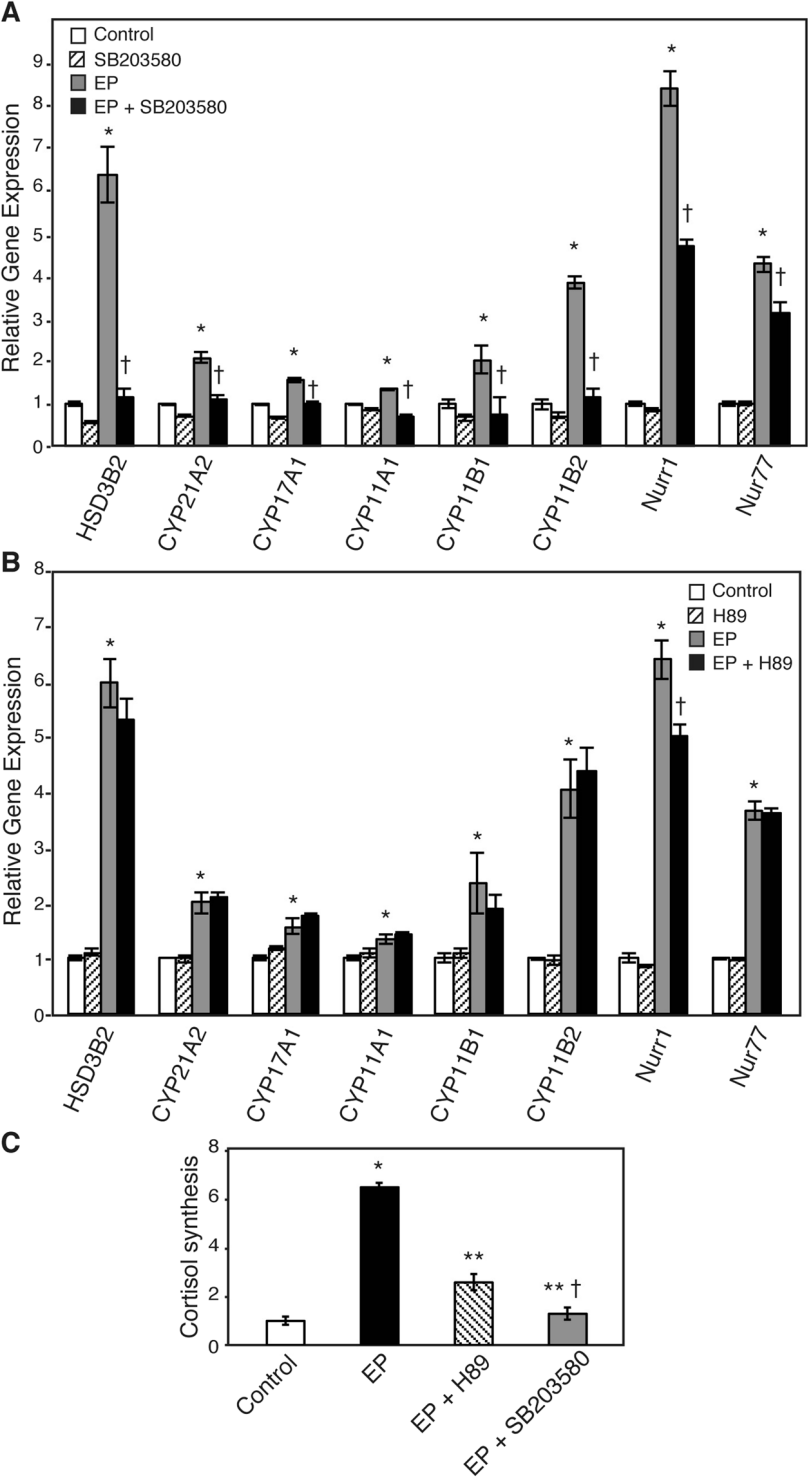
Effects of a specific p38MAPK inhibitor and specific PKA inhibitor on steroidogenesis. H295R cells were treated with EP (0.75 μM) for 72 h, changed to the normal growth medium containing the p38MAPK inhibitor SB203580 (10 μM) (**A**) or PKA inhibitor H89 (10 μM) (**B**), and cultured for another 24 h. (**A**,**B**) Relative mRNA expression of the indicated genes was analyzed by RT-qPCR. Data are presented as the mean ± SE of 3 independent experiments. *P < 0.05 vs. control, ^†^P < 0.05 vs. EP. (**C**) Cortisol concentration in the medium was measured using ELISA and corrected by the number of cells. Data are presented as the mean ± SE of 3 independent experiments. *P < 0.005 vs. control, **P < 0.005 vs. EP, and ^†^P < 0.005 vs. EP + H89.

As p38MAPK is known as one of the main downstream molecules of GADD45A, the effect of siGADD45A on the phosphorylation level of p38MAPK was analyzed. Western blotting of p38MAPK showed that the level of phosphorylated p38MAPK, normalized by p38MAPK, was increased by approximately 2-fold in EP-treated cells, and decreased to control level by treatment with siGADD45A, suggesting that EP induced the phosphorylation of p38MAPK via GADD45A utilization (Fig. 4A and B). RT-qPCR revealed that SB203580, an inhibitor of p38MAPK, significantly reduced the levels of HSD3B2, CYP21A2, CYP17A1, CYP11A1, CYP11B1, CYP11B2, Nurr1, and Nur77 mRNA levels (Fig. 5A and Supplementary Table S6).

During acute stress, the activation of the HPA axis promotes steroidogenesis in adrenocortical cells by increasing the concentration of c-AMP, followed by PKA activation. Therefore, we explored whether EP-induced steroidogenesis is involved in PKA activation. H89, an inhibitor of PKA, significantly decreased the expression of Nurr1 mRNA by 22% in EP-treated cells (Fig. 5B and Supplementary Table S7). The other factors examined were not changed. EP-induced cortisol synthesis was decreased to 40% or 20% by H89 or SB203580, respectively (Fig. 5C). SB203580 was significantly more effective than H89 at suppressing EP-induced cortisol synthesis. On the other hand, 8-bromo-cAMP (8-Br-cAMP), an analog of cAMP, remarkably increased the expression of steroidogenesis-related genes, but not GADD45A (Supplementary Figs S4 and S5). Furthermore, H89 significantly decreased HSD3B2, CYP17A1, CYP11B1, CYP11B2, and Nurr1 expression (Supplementary Fig. S4). siGADD45A did not reduce the level of those steroidogenesis-related genes in 8-Br-cAMP-stimulated cells (Supplementary Fig. S5). These data suggest that EP-induced steroidogenesis is mainly facilitated by GADD45A-p38 MAPK pathway, while 8-Br-cAMP-induced steroidogenesis is regulated by PKA, supporting our concept that EP-induced steroidogenesis occurs via a mechanism distinct from the HPA axis.

## Discussion

In this study, we found that EP, which induces the DDR, promoted steroidogenesis in human adrenal H295R cells in a cell-autonomous manner, accompanied by the upregulation of steroidogenesis-related genes expression and the accumulation of CYP21A2 in particular, leading to significant cortisol production. The induction of steroidogenesis occurred through GADD45A upregulation and p38MAPK activation.

EP is a chemotherapeutic agent that induces DSBs by inhibiting DNA topoisomerase II (TopII)^59^. A high or low dose of EP induces apoptosis or cellular senescence, respectively^60^. The induction of DSBs triggers the phosphorylation of one of the variants of the nucleosome core histone H2A, namely, histone H2AX at Ser^139^ (γH2AX). This phosphorylation, which is mediated by ATM, ATR, and/or DNA-dependent protein kinases, takes place in nucleosomes along a megabase of DNA flanking the DSB. As the immunocytochemical detection of γH2AX foci reveals that DNA damage with DSBs has occurred in cells, γH2AX is considered a biomarker of DNA damage. In this study, we treated H295R cells with a low dose of EP, which did not induce apoptosis. From 3 days after EP-treatment and thereafter, γH2AX foci-positive cells were increased significantly, confirming that EP induces DNA damage in H295R cells. At the same time, the accumulation of CYP21A2 and the expression of steroidogenesis-related genes were induced. Double staining of γH2AX and CYP21A2 showed the tight relationship between DNA damage and the induction of the steroidogenic enzyme CYP21A2 (Fig. 1A–D and Supplementary Table S1). These results indicate that steroidogenesis is activated in H295R cells undergoing the DDR without the aid of other stimuli. H295R cells are the only human adrenocortical cell line that has the potential to produce steroid hormones, mineralocorticoids, glucocorticoids, and adrenal androgens in a physiological manner^61^. There are two available and well-characterized adrenocortical cell lines that have the ability to produce steroid hormones, H295R cells and Y1 mouse adrenocortical tumor cells. We treated Y1 cells with EP and analyzed steroidogenesis. As Y1 cells do not produce corticosterone^56,62^, we assessed progesterone secretion in response to EP. As shown in Supplementary Fig. S6A and B, we observed a 2.5-fold increase in progesterone secretion, which was associated with the upregulation of StAR, CYP11A1, and CYP11B1 gene expression. As limited steroid hormone production is life-threatening, the induction of steroidogenesis in DNA-damaged adrenocortical cells might be essential for survival *in vivo*. We suspect that the activation of steroidogenesis in response to DNA damage might be a general mechanism in mammalian adrenocortical cells.

In this study, we found that GADD45A expression was upregulated, and knockdown of GADD45A using siRNA inhibited EP-induced steroidogenesis, which was accompanied by blocking the expression of steroidogenesis-related genes, such as HSD3B2 (Figs 2 and S3). Further, the transient overexpression of GADD45A in H295R cells upregulated the expression of HSD3B2 and CYP21A2 mRNA, which was accompanied with CYP21A2 protein expression (Fig. 3). These findings indicate that GADD45A plays an important role in EP-induced steroidogenesis. By contrast, 8-Br-cAMP-induced steroidogenesis was not inhibited by siGADD45A (Supplementary Fig. S5). These data indicate that GADD45A is associated specifically with EP-induced steroidogenesis. GADD45A protein lacks enzymatic activity and exerts its function by interacting with effectors of the cell cycle, apoptosis, DNA repair, and cellular senescence, and decides cell fates. In this study, an inhibitor of p38MAPK inhibited EP-induced steroidogenesis (Fig. 5A and C), suggesting that p38MAPK is involved in this process. Several papers reported that GADD45A binds to and activates p38MAPK^40–42^, or MAP3K4, which is known as an upstream kinase of p38MAPK^55,63–65^. Burllard *et al*. reported that GADD45A activates MEKK4 by forming a complex, which is followed by the induction of skeletal muscle fiber atrophy^65^. GADD45A might induce steroidogenesis by interacting with MEKK4. On the other hand, GADD45A expression was not increased in EP-treated Y1 cells (Supplementary Fig. S6B). We revealed that StAR gene expression was upregulated by EP (Supplementary Fig. S6B). It is reported that KRAS is expressed at a high level in Y1 cells^56^. Thus, a mechanism other than the induction of GADD45A might underlie EP-induced steroidogenesis in Y1 cells.

We demonstrated that siGADD45A reduced p38MAPK phosphorylation (Fig. 4). An inhibitor of p38MAPK, SB203580, reduced the EP-induced upregulation of steroidogenesis-related gene expression and glucocorticoid production (Fig. 5A and C). These data suggest that p38MAPK is located downstream of GADD45A in EP-induced steroidogenesis. It is important to identify the target(s) of p38MAPK to clarify the mechanism of EP-induced steroidogenesis. p38MAPK phosphorylates several major transcriptional factors, such as CREB and GATA. There are GATA binding sites in the promoter of HSD3B2^66^, while CREB binding sites are present in the promoters of Nurr1, Nur77, CYP11B1, and CYP11B2^67–69^. Thus, it seems likely that p38MAPK regulates steroidogenesis-related gene expression and glucocorticoid production. The identification of factors connecting p38MAPK to steroidogenesis is an important aim of a future study.

The mammalian stress response involves the activation of the HPA axis, followed by an increase of cAMP and PKA activation, resulting in steroidogenesis in adrenocortical cells through the upregulation of SF1 transcripts. In this study, the steroidogenesis induced by EP via the DDR was partially inhibited by the PKA inhibitor H89 (Fig. 5B and C), while the overall steroidogenesis induced by 8-Br-cAMP, a cAMP analog and PKA activator, was effectively inhibited by H89 (Supplementary Fig. S4). Of particular interest, SF1 and StAR gene expression was not affected in EP-treated cells (Supplementary Fig. S2). These data strongly indicate that the DDR may surely promote steroidogenesis via a pathway other than the HPA axis.

The HPA axis is self-regulated by a negative feedback exerted by serum cortisol levels in both the hypothalamus and the pituitary gland. As EP-induced steroidogenesis is independent of the HPA axis, the production of too much glucocorticoid cannot be self-regulated by the normal negative feedback mechanism. For example, various degrees of DNA damage occur in aged organs, and it has been reported that glucocorticoid concentration is elevated in the serum of aged mice^22^. Age-related increases in cortisol production have been documented in human using blood and salivary samples^23–26^. Several papers reported that a decrease in the sensitivity of the HPA axis to glucocorticoid feedback suppression occurs with aging^70–75^. The increases in cortisol observed with aging have been attributed to an impairment of feedback inhibition of HPA activity due to neuronal loss in the hippocampal area^23,25^. Thus, our present study might suggest that such an elevation of glucocorticoid may originate from DNA damage in aged adrenal glands through the upregulation of the stress-associated protein GADD45A and the activation of p38MAPK. The elucidation of the mechanism of DNA damage-induced glucocorticoid production, leading to prohibition of glucocorticoid excess caused by DNA damage, may provide a new approach toward the prevention of some of the complications associated with aging and age-related diseases.

In this study, we treated H295R cells with a low dose of EP, which induced DNA damage, to mimic cellular senescence. We examined the expression of certain factors of the senescence-associated secretary phenotype (SASP) and senescence marker p16^Ink4^ (p16). As shown in Supplementary Fig. S7A, at 96 h, a significant upregulation of IL8 and MMP10 mRNA was observed (IL8: 7.0-fold, MMP10: 3-fold). Thereafter, at 7 days and 20 days after EP treatment, the expression decreased to the control level. On the other hand, p16 remained unchanged throughout. The cell cycle analysis by flow cytometry revealed that the population of cells in the G2/M phase was significantly increased following EP treatement, and was decreased to the control level at 20 days after EP treatment (Supplementary Fig. S7B). These findings indicate that the upregulation of IL8 and MMP10 and cell cycle inhibition in the G2/M transition occurred temporally and thereafter recovered. These data might suggest that the cells were immortalized and not induced to be fully senescent by EP treatment. Indeed, it may be difficult to induce cellular senescence in H295R cells. It has been reported that H295R cells lack telomerase activity, but maintains their telomeres by the alternative lengthening of telomeres (ALT) mechanism^76^. Further, Sampaoli C, *et al*. reported that H295R cells have p53 mutation, the lack of exons 8 and 9, including the part of the DNA binding domain and the entire C-terminal domain^77,78^, suggesting that p53 activity is completely lost. Ragazzon B *et al*. reported that H295R cells carry a homozygous deletion of the RB transcriptional corepressor 1 (RB1) gene^79^, and Hadjadj *et al*. reported that RB is not detected in H295R cells^80^. Indeed, we did not detect exon 8–9 of p53 or whole RB1 in these cells as shown in Supplementary Fig. S8. Thus cellular senescence might barely occur in H295R cells, due to the lack of p53 activity and RB pathways as well as the presence of the ALT pathway. Although the present study may not represent a typical model that uses an aged adrenal gland, an increase in the glucocorticoid production was one of the features of adrenal glands in aging mammals^22–26^.

In summary, we showed that EP activates cell-autonomous steroidogenesis in H295R cells undergoing DNA damage, which may constitute a part of senescence, through the expression of genes for steroidogenic factors and enzymes. The EP-induced upregulation of the stress-associated protein GADD45A and activation of p38MAPK were followed by steroidogenesis. This study provides novel information on one of the diverse mechanisms of steroidogenesis associated with aging.

## Methods

### Materials

EP and 8-Br-cAMP were purchased from Sigma-Aldrich (St. Louis, MO, USA). SB203580 and H89 were purchased from Cayman Chemical Company (Ann Arbor, MI, USA) and EMD Millipore (Temecula, CA, USA), respectively. The following antibodies were used in this study: primary antibodies: anti-mouse FLAG M2 monoclonal (F1804; Sigma-Aldrich), anti-mouse γH2AX monoclonal (05-636; EMD Millipore), anti-rabbit p38MAPK polyclonal (#9212; CST, Danvers, MA, USA), anti-rabbit GADD45A polyclonal (sc-792; Santa Cruz Biotechnology, Dallas, TX, USA), anti-rabbit phospho-p38MAPK (Thr180–Tyr182) polyclonal (#9211; CST), and anti-goat CYP21A2 polyclonal (C-17) (sc-48466, Santa Cruz, Santa Cruz, CA, USA); and secondary antibodies: Alexa Fluor 647 goat anti-mouse/rabbit IgG (H + L) (A-21236/A-21245; Molecular Probes, Eugene, OR, USA), Alexa Fluor 647 donkey anti-goat IgG (H + L) (A-21447; Molecular Probes), Alexa Fluor 546 goat anti-mouse IgG (H + L) (A-11030, Molecular Probes), and Alexa Fluor 546 donkey anti-mouse/rabbit IgG (A-10036/A-10040; Molecular Probes).

### Cell culture and treatment

Human adrenal cortical cells (NCI-H295R pluripotent adrenocortical carcinoma cell line) were purchased from the American Type Culture Collection (ATCC, Manassas, VA, USA). The cells were cultured in the normal growth medium, DMEM/F-12K (1:1) (ATCC) supplemented with 1% ITS + Premix (final concentrations of 6.25 μg/mL insulin/transferrin, 6.25 ng/mL selenium, 1.25 mg/mL bovine serum albumin, and 5.35 μg/mLlinoleic acid) (BD Biosciences, Bedford, MA, USA), 2.5% NuSerum (NuSerum containing 25% new born calf serum) (BD Biosciences), and 100 U/mL penicillin/0.1 mg/mL streptomycin (GIBCO BRL, Palo Alto, CA, USA), at 37 °C in a humidified atmosphere containing 5% CO_2_^57^. The cells for experiments were plated in 6-well plates at a density of 6.0 × 10^5^ cells per well and cultured in the normal growth medium for 24 h.

### EP treatment

At 1 day after plating, the cells were treated with EP for 72 h, changed to the normal growth medium, and cultured for another 24 h (96 h after treatment). RNA was extracted from the cells at 72 h and 96 h after EP treatment. For western blotting and immunofluorescence, the cells were harvested at 96 h. The culture medium was removed from the cells at 96 h and stored at −80 °C until assayed for cortisol by a radioimmunoassay.

### 8-Br-cAMP treatment

At 1 day after plating, the cells were treated with 8-Br-cAMP (500 μM) for 24 h, and then, harvested for RNA.

### Inhibitors treatment

The p38MAPK inhibitor, SB203580 (10 μM) and PKA inhibitor, H89 (10 μM) were added at 24 h before harvest.

### Transient transfection

At 1 day after plating, pcDNA3.1Zeo (+) (empty vector) or pCMV3-human GADD45A-C-FLAG vector (Sino Biological, Inc., Beijing, China) was introduced into H295R cells using Lipofectamine 2000 Reagent (Invitrogen, Carlsbad, CA, USA). At 2 days after transfection, the cells were harvested for RNA or fixed with 4% paraformaldehyde for immunofluorescence.

### siRNA transfection

Control siRNA was obtained from Dharmacon (Thermo Fisher Scientific, Inc., Waltham, MA, USA). siRNAs for GADD45A were obtained from Dharmacon (Thermo Fisher Scientific, Inc.) and Invitrogen (Thermo Fisher Scientific, Inc.) (see Supplementary Materials and Methods for details of siRNAs). siRNAs were transfected into the cells using RNAiMAX (Invitrogen/ThermoFisher Science, Inc.), according to the manufacturer’s reverse transfection method at the same time as plating.

### Western blotting

The cells were harvested by scraping into phosphate-buffered saline (PBS) and collected by centrifugation. The cells were lysed in approximately 10 pellet volumes of sample buffer (60 mM Tris pH 6.8, 2% SDS, 10% glycerol, 100 mM DTT) and boiled for 5 min. Whole cell extracts were standardized for protein content using a Protein Assay Kit (Bio-Rad, Hercules, CA, USA). Cell extracts (20 μg protein) were separated by SDS-PAGE and transferred onto a PVDF membrane (Cat. 10600022; Amersham; Little Chalfont, UK) using the wet-type transfer method. The membranes were blocked for 1 h with 5% non-fat dry milk. Then, the blots were incubated overnight at 4 °C with primary antibodies (anti-p38MAPK antibody, 1:1000 in 5% milk/TTBS; anti-phospho-p38MAPK antibody, 1:1000 in 5% milk/TTBS). The membranes were incubated with the secondary antibodies for 1 h at room temperature. After the final washes, the membranes were scanned using Typhoon Trio (Amersham), and the signals of reactive bands were quantified using ImageJ software.

### Cortisol assays

Cortisol production from H295R cells was analyzed by measuring its concentration in culture medium using a Chemiluminescent Enzyme Immunoassay (LSI Medience Corporation, Tokyo, Japan), and normalized by cell number. Three independent experiments were performed in triplicate, and followed by statistical analyses.

### Reverse-transcription and real-time quantitative PCR (RT-qPCR)

Total RNA was extracted with RNA iso-Plus (TaKaRa Bio, Inc., Shiga, Japan) according to the manufacturer’s instructions. Total RNA (1 μg) was reverse-transcribed to cDNA using a PrimeScript RT Reagent Kit with gDNA Eraser (RR047A, TaKaRa) according to the manufacturer’s instructions. qRT-PCR was performed using a One Step SYBR PrimeScript RT-PCR Kit Ver. 1 (RR066A; TaKaRa) and a Thermal Cycler Dice Real-Time System (TP800; TaKaRa) according to the manufacturer’s instructions. The thermal cycling conditions consisted of an initial denaturation step at 95 °C for 30 s followed by 40 cycles of PCR under the following conditions: 95 °C for 5 s and 60 °C for 60 s. *GAPDH* was used as an internal control because this gene, along with the cyclophilin gene, is employed widely as an internal control for the changes of mRNA levels of steroidogenic enzyme genes^57,81,82^. Our choice of appropriately distant primer sets and experiments with or without reverse transcriptase excluded the possibility that our real-time RNA quantification counted genomic DNA. The relative amount of each transcript was calculated with the 2^−∆∆Ct^ method^83^ using the cycle threshold value, which was determined automatically by the real-time PCR system by means of the second derivative maximum method^84^. Primer pairs were subsequently tested for performance: absence of primer dimers and efficiency of amplification >95%, <105%. The primer sets are described in Table S8. Three independent experiments were performed in triplicate, and followed by statistical analyses.

### Immunofluorescence analysis

The cells were cultured on coverslips in 6-well plates. The cells were fixed with 4% paraformaldehyde and 4% sucrose in PBS for 20 min at room temperature. Permeabilization was carried out with 0.2% Triton X-100 in PBS for 20 min at room temperature. Nonspecific binding was blocked by incubation in 3% bovine serum albumin and 0.1% Triton X-100 in PBS for 30 min at 37 °C. The antibodies were diluted in the above blocking solution at the indicated concentrations and incubated for O/N at 4 °C. Secondary antibodies were also diluted in the blocking solution and incubated for 1 h at room temperature. Nuclei were stained with 2 μg/mL 4′6-diamidine-2′-phenylindole dihydrochloride (DAPI) (Roche, Mannheim, Germany) in PBS for 15 min at room temperature. Images were acquired using a laser-scanning confocal image system (A1R-A1 Confocal Microscope System; Nikon, Tokyo, Japan). The primary anti-flag, anti-γH2AX, anti-GADD45A, and anti-CYP21A2 antibodies were used at concentrations of 1:1000, 1:50, 1:250, and 1:50, respectively, and the secondary antibodies were used at a concentration of 1:500.

### Statistical analysis

Quantitative data are expressed as the mean ± standard error (SE). Statistical analysis was performed by Student’s *t* test or one-way analysis of variance followed by a post-hoc Turkey’s test using JMP9 software.

## Electronic supplementary material

Supplementary Information
