# Supplementary material for: DNA damage response induced by Etoposide promotes steroidogenesis via GADD45A in cultured adrenal cells
Source: Sci Rep. 2018 Jun 25;8:9636. doi: 10.1038/s41598-018-27938-5 (PMC6018231; doi:10.1038/s41598-018-27938-5)

**DNA damage response induced by Etoposide promotes steroidogenesis via Gadd45a in cultured adrenal cells**

Mimi Tamamori-Adachi[*1], Akane Koga[1,2], Takao Susa[1], Hiroko Fujii[1,3], Masao Tsuchiya[2], Hiroko Okinaga[4], Harumi Hisaki[1], Masayoshi Iizuka[1], Shigetaka Kitajima[5] and Tomoki Okazaki[1]

[1]Department of Biochemistry, [4]Department of Internal Medicine, Teikyo University School of Medicine, [2]Department of Practical Pharmacy, Faculty of Pharmaceutical Sciences, Teikyo University, 2-11-1, Kaga, Itabashi-ku, Tokyo, 173-8605, Japan

[3]Department of General Medicine, National Defense Medical College, 3-2, Namiki, Tokorozawa City, Saitama, 359-8513, Japan

[5]Department of Biochemical Genetics, Medical Research Institute, Tokyo Medical and Dental University, 1-5-45, Yushima, Bunkyo-ku, Tokyo, 113-8605, Japan

*Address Correspondence to: Mimi Tamamori-Adachi, MD, PhD

Department of Biochemistry, Teikyo University School of Medicine, 2-11-1, Kaga, Itabashi-ku, Tokyo, 173-8605, Japan; Tel: +81-3-3964-3649; Fax: +81-3-5375-6366

E-mail: madachi@med.teikyo-u.ac.jp

**Supplementary Information: Supplementary Materials and Methods, Supplementary References, Table S1-S9, and Figure S1-S9**

**Supplementary Materials and Methods**

***Y1 mouse adrenocortical cell culture and treatment***

Y1 mouse adrenocortical cells were purchased from the JCRB Cell Bank (National Institutes of Biomedical Innovation, Health and Nutrition, Osaka, Japan). The cells were cultured in Ham's F10 (GIBCO, Thermofisher Scientific Inc, Waltham, MA, USA) supplemented with 2.5% of FBS (ATCC), 15% of Horse serum (GIBCO, Thermofisher Scientific Inc), L-glutamine (GIBCO, Thermofisher Scientific Inc), and 100 U/mL penicillin/0.1 mg/mL streptomycin (GIBCO, Thermofisher Scientific Inc), at 37 °C in a humidified atmosphere containing 5% $CO_2$. The cells for experiments were plated in 6-well plates at a density of $6.0 \times 10^5$ cells per well and cultured for 72 h. At 72 h after plating, the cells were treated with EP for 72 h, changed with the normal growth medium, and cultured for another 24 h. The cells were harvested for RNA for qRT-PCR, or fixed for FACS.

***LNCaP cells culture***

Prostate cancer LNCaP cells were cultured as described previously (Susa et al., 2015). The cells were cultured in RPMI-1640 medium (GIBCO, Thermofisher Scientific Inc), supplemented with 5% (v/v) fetal bovine serum (GIBCO, Thermofisher Scientific Inc) and antibiotics (GIBCO, Thermofisher Scientific Inc) in humidified 5% $CO_2$-95% air at 37°C.

***Progesterone assay***

Progesterone production from Y1 cells was analyzed by measuring its concentration in culture medium using LC-MS/MS (ASAKA Pharma Medical Co. Ltd., Kanagawa, Japan) (Kobayashi et al, Scientific Reports, 2016) , and normalized by cell number. Three independent experiments were performed in triplicate, and followed by statistical analyses.

***Cell cycle analysis by flow cytometry***

Cells were harvested by trypsinization, fixed with 70% ethanol at 4 °C, incubated with 1 μg /ml RNaseA in PBS for 30 min at room temperature, and stained with 50 μg /ml propidium iodide for 1 hr at room temperature. Analysis was performed using a BD FACSCantoTM II flow cytometer (BD Biosciences, Franklin Lakes, NJ).

***siRNAs for GADD45A***

For Figure 2, siRNAs for the GADD45A obtained from Dharmacon/Thermo Fisher Scientific, Inc. Those are mixed of 4 kinds of siGENOME SMARTpool siRNA (D-003893-02, GADD45A, Target Sequence: GATCCTGCCTTAAGTCAAC), (D-003893-04, GADD45A, Target Sequence: CCGAAAGGATGGATAAGGT), (D-003893-05, GADD45A, Target Sequence: CTACATGGATCAATGGGTT), and (D-003893-06, GADD45A, Target Sequence: TAATCTCCCTGAACGGTGA). siGADD45A-2 for Supplementary Figure S2 (Stealth RNAi; cat. no. 10620318-350610-G05 and 10620319-350610 E09, Target sequences:

GGTGACGAATCCACATTCATCTCAA) were purchased from Invitrogen (Thermo Fisher Scientific, Inc.).

**Supplementary References**

Susa, T. *et al.* Wild-type and specific mutant androgen receptor mediates transcription via 17beta-estradiol in sex hormone-sensitive cancer cells. *J. Cell. Physiol.* **230**, 1594-1606 (2015).

Kobayashi, M. *et al.* Sex differences in the serum level of endogenous ligands for estrogen receptorβ in the elderly population. *Sci. Rep.* **6,** 25878 (2016)

Chappel, W. H. *et al.* p53 expression controls prostate cancer sensitivity to chemotherapy and the MDM2 inhibitor Nutlin-3. *Cell Cycle* **11,** 4579-4588 (2012).

Sharma, A. *et al.* Retinoblastoma tumor suppressor status is a critical determinant of therapeutic response in prostate cancer cells. *Cancer Res.* **67,** 6192-6203 (2007).

**Table S1, Related to Figure 1D**

| population | Total | Total | Total | Total | γH2AX (+) | γH2AX (-) |
|---|---|---|---|---|---|---|
| γH2AX | (+) | (+) (-) | (+) | (-) | (+) | (-) |
| CYP21A2 | (+) (-) | (+) | (+) | (-) | (+) | (+) |
| Control | 9.1±7.42 | 8.9±1.2 | 0±0 | 82±2.86 | 0±0 | 9.8±1.49 |
| EP | 63.4±7.43* | 26.6±0.45* | 22.7±1.43* | 32.7±6.43* | 36.3±2.0* | 10.3±0.97 |

**The percentages of γH2AX-, or CYP21A2-, positive, or negative cells in total cells, or γH2AX-positive, or -negative cells**

H295R cells were treated with EP (0.75 μM) for 72 h, changed with the same growth medium, and cultured for another 24 h, and fixed with 4% paraformaldehyde, followed by immunofluorescence. The percentages of γH2AX, or CYP21A2, -positive or -negative cells in total, or γH2AX-positive or -negative cells were measured. At least 200 cells from random fields in a blinded manner were scored for each condition. Data are presented as mean ± SE of three independent experiments. *P < 0.05 vs. control.

**Table S2, Related to Figure 1F**

| | control | 72 h | 96 h |
|---|---|---|---|
| HSD3B2 | 1.00 ± 0.082 | 3.92 ± 0.177* | 5.82 ± 0.527* |
| CYP21A2 | 1.00 ± 0.015 | 1.47 ± 0.062* | 2.17 ± 0.247* |
| CYP17A1 | 1.00 ± 0.030 | 1.5 ± 0.079* | 1.69 ± 0.057* |
| CYP11A1 | 1.00 ± 0.104 | 1.28 ± 0.093* | 1.42 ± 0.060* |
| CYP11B1 | 1.00 ± 0.134 | 1.18 ± 0.079 | 2.21 ± 0.299* |
| CYP11B2 | 1.00 ± 0.084 | 2.64 ± 0.173* | 3.69 ± 0.344* |
| Nurr1 | 1.00 ± 0.075 | 1.81 ± 0.078* | 6.85 ± 0.583* |
| Nur77 | 1.00 ± 0.145 | 1.63 ± 0.083* | 4.50 ± 0.130* |
| GADD45A | 1.00 ± 0.174 | 1.61 ± 0.092* | 3.79 ± 0.413* |

**Fold change of mRNA expression of the indicated genes**

H295R cells were treated with EP (0.75 μM) for 72 h (72h), changed to the same growth medium, and cultured for another 24 h (96h). Data are presented as the mean ± SE of 3 independent experiments. *P < 0.05 vs. control.

**Table S3, Related to Figure 2C**

| | siControl | siGADD45A | EP + siControl | EP + siGADD45A |
|---|---|---|---|---|
| HSD3B2 | 1.00 ± 0.088 | 0.82 ± 0.030 | 4.66 ± 0.161* | 2.62 ± 0.064† |
| CYP21A2 | 1.00 ± 0.062 | 0.98 ± 0.005 | 2.13 ± 0.180* | 1.38 ± 0.099† |
| CYP17A1 | 1.00 ± 0.014 | 1.01 ± 0.007 | 2.32 ± 0.200* | 1.57 ± 0.074† |
| CYP11A1 | 1.00 ± 0.028 | 0.96 ± 0.021 | 1.70 ± 0.106* | 1.39 ± 0.143† |
| CYP11B1 | 1.00 ± 0.086 | 0.87 ± 0.013 | 2.75 ± 0.490* | 1.23 ± 0.187† |
| CYP11B2 | 1.00 ± 0.160 | 1.09 ± 0.006 | 3.45 ± 0.117* | 1.27 ± 0.130† |
| Nurr1 | 1.00 ± 0.058 | 1.30 ± 0.020 | 2.82 ± 0.127* | 2.13 ± 0.066† |
| Nur77 | 1.00 ± 0.027 | 1.14 ± 0.035 | 2.89 ± 0.265* | 1.92 ± 0.039† |
| GADD45A | 1.00 ± 0.067 | 0.75 ± 0.027 | 3.48 ± 0.205* | 1.42 ± 0.024† |

**Fold change of mRNA expression of the indicated genes**

H295R cells were transfected with the indicated siRNAs. After 24 h, the cells were treated with EP (0.75 μM) for 72 h, changed to the normal growth medium, and cultured for another 12 h. Relative mRNA expression of the indicated genes was analyzed by qRT-PCR. Data are presented as the mean ± SE of 3 independent experiments. *P < 0.05 vs. control, †P < 0.05 vs. EP + siControl.

**Table S4, Related Figure 3A**

| | Empty | GADD45A |
|---|---|---|
| HSD3B2 | 1.00 ± 0.074 | 1.67 ± 0.130* |
| CYP21A2 | 1.00 ± 0.053 | 1.93 ± 0.105* |
| CYP11B1 | 1.00 ± 0.027 | 1.42 ± 0.170* |
| CYP11B2 | 1.00 ± 0.049 | 1.93 ± 0.154* |
| Nurr1 | 1.00 ± 0.042 | 1.38 ± 0.068* |

**Fold change of mRNA expression of the indicated genes**

H295R cells were transiently transfected with the empty vector or human GADD45A expression vector, and cultured for another 48 h. Relative mRNA expression of the indicated genes was analyzed by qRT-PCR. Data are presented as the mean ± SE of 3 independent experiments. *$P < 0.05$ vs. empty.

**Table S5, Related to Figure 3C**

| Transfection | Empty | FLAG-GADD45A | | |
|---|---|---|---|---|
| | Total | Total | FLAG (+) | FLAG (-) |
| CYP21A2 (+) (%) | 9.5±1.22 | 12.5±2.24 | 55.9±7.1$^{*, **, ***}$ | 10.9±1.87 |

**The percentages of CYP21A2-positive cells in total cells, or FLAG-positive or -negative cells**

H295R cells were transiently transfected with the empty vector or human GADD45A expression vector, and cultured for another 48 h, followed by fixation with 4% paraformaldehyde and immunofluorescence with anti-FLAG and anti-CYP21A2 antibodies.

The percentages of steroidogenic CYP21A2-positive cells in empty vector-transfected cells (Empty), total FLAG-GADD45A transfected cells (Total), FLAG-GADD45A transfected FLAG-positive [FLAG (+)] or -negative [FLAG (-)] cells were measured.

At least 200 cells from random fields in a blinded manner were each condition. Data are presented as mean ± SE of three independent experiments. *P < 0.05 vs. Empty, **P < 0.05 vs. Total, ***P < 0.05 vs. FLAG (-).

**Table S6, Related to Figure 5A**

| | Control | SB203580 | EP | EP + SB203580 |
|---|---|---|---|---|
| HSD3B2 | 1.00 ± 0.065 | 0.57 ± 0.042 | 6.38 ± 0.661* | 1.18 ± 0.188† |
| CYP21A2 | 1.00 ± 0.022 | 0.73 ± 0.021 | 2.08 ± 0.124* | 1.09 ± 0.135† |
| CYP17A1 | 1.00 ± 0.022 | 0.69 ± 0.027 | 1.57 ± 0.064* | 0.99 ± 0.067† |
| CYP11A1 | 1.00 ± 0.018 | 0.88 ± 0.030 | 1.35 ± 0.023* | 0.69 ± 0.084† |
| CYP11B1 | 1.00 ± 0.111 | 0.69 ± 0.082 | 2.04 ± 0.319* | 0.77 ± 0.406† |
| CYP11B2 | 1.00 ± 0.123 | 0.73 ± 0.053 | 3.85 ± 0.145* | 1.16 ± 0.190† |
| Nurr1 | 1.00 ± 0.053 | 0.86 ± 0.029 | 8.37 ± 0.406* | 4.68 ± 0.168† |
| Nur77 | 1.00 ± 0.042 | 1.00 ± 0.030 | 4.27 ± 0.177* | 3.12 ± 0.265† |

**Fold change of mRNA expression of the indicated genes**

H295R cells were treated with EP (0.75 μM) for 72 h, changed with the normal growth medium containing the p38MAPK inhibitor SB203580 (10 μM), and cultured for another 24 h. Relative mRNA expression of the indicated genes was analyzed by qRT-PCR. Data are presented as the mean ± SE of 3 independent experiments. *$P < 0.05$ vs. control, †$P < 0.05$ vs. EP.

**Table S7, Related to Figure 5B**

| | Control | H89 | EP | EP + H89 |
|---|---|---|---|---|
| HSD3B2 | 1.00 ± 0.032 | 1.10 ± 0.069 | 5.97 ± 0.444* | 5.33 ± 0.364 |
| CYP21A2 | 1.00 ± 0.009 | 0.99 ± 0.069 | 2.01 ± 0.203* | 2.10 ± 0.102 |
| CYP17A1 | 1.00 ± 0.040 | 1.18 ± 0.029 | 1.57 ± 0.133* | 1.77 ± 0.041 |
| CYP11A1 | 1.00 ± 0.040 | 1.10 ± 0.081 | 1.35 ± 0.090* | 1.42 ± 0.065 |
| CYP11B1 | 1.00 ± 0.079 | 1.09 ± 0.096 | 2.36 ± 0.552* | 1.91 ± 0.238 |
| CYP11B2 | 1.00 ± 0.026 | 0.97 ± 0.088 | 4.05 ± 0.533* | 4.37 ± 0.431 |
| Nurr1 | 1.00 ± 0.082 | 0.87 ± 0.033 | 6.38 ± 0.340* | 4.99 ± 0.216[†] |
| Nur77 | 1.00 ± 0.018 | 0.99 ± 0.011 | 3.68 ± 0.173* | 3.61 ± 0.094 |

**Fold change of mRNA expression of the indicated genes**

H295R cells were treated with EP (0.75 μM) for 72 h, changed to the normal growth medium containing the PKA inhibitor H89 (10 μM), and cultured for another 24 h. Relative mRNA expression of the indicated genes was analyzed by qRT-PCR. Data are presented as the mean ± SE of 3 independent experiments. *$P < 0.05$ vs. control, †$P < 0.05$ vs. EP.

**Table S8, Human primer sequences**

| Gene | | Primer sequence |
|---|---|---|
| HSD3β2 | Forward | 5' -GCCTGTTGGTGGAAGAGAAG- 3' |
| | Reverse | 5' -ATGATACAGGCGGTGTGGAT- 3' |
| CYP21A2 | Forward | 5' -AACTACCCGGACCTGTCCTT- 3' |
| | Reverse | 5' -TCTCATGCGCTCACAGAACT- 3' |
| CYP17A1 | Forward | 5' -CTCTCTACTCGGTTCTCGGC- 3' |
| | Reverse | 5' -GGACACCTTGCCCACATCT- 3' |
| CYP11A1 | Forward | 5' -GGAAATTACTCGGGGGACAT- 3' |
| | Reverse | 5' -CACATGGTCCTTCCAGGTCT- 3' |
| CYP11B1 | Forward | 5' -AGGAGACCTTGCGGCTCTACC- 3' |
| | Reverse | 5' -GAACACGCGCACCAATGTC- 3' |
| CYP11B2 | Forward | 5' -CCCCATGAAAGGGGAAATAC- 3' |
| | Reverse | 5' -GTTGCCCCCTTATTCCTTTC- 3' |
| Nurr1 | Forward | 5' -AGGCTTCTTTAAGCGCACAG- 3' |
| | Reverse | 5' -TCTTTGACCATCCCAACAGC- 3' |
| Nur77 | Forward | 5' -CAGTGCAGAAAAACGCCAAG- 3' |
| | Reverse | 5' -TTCGGACAACTTCCTTCACC- 3' |
| SF1 | Forward | 5' -AAGGTGTCCGGCTACCACTA- 3' |
| | Reverse | 5' -CTTGTACATCGGCCCAAACT- 3' |
| StAR | Forward | 5' -GCATCGGTGAGTTTGCTGTG- 3' |
| | Reverse | 5' -GATTCAAGAAACGCTCAGC- 3' |
| GADD45A | Forward | 5' -AGCAGAAGACCGAAAGGATG- 3' |
| | Reverse | 5' -AGGCACAACACCACGTTATC- 3' |
| IL8 | Forward | 5' -AAGAAACCACCGGAAGGAAC- 3' |
| | Reverse | 5' -ACTCCTTGGCAAAACTGCAC- 3' |
| MMP10 | Forward | 5' -TTTTGGCGAAGATCCCACTG- 3' |
| | Reverse | 5' -TAAAAACGGTGTCCCTGCTG- 3' |
| p16 | Forward | 5' -CGGAAGGTCCCTCAGACATC- 3' |
| | Reverse | 5' -CCCTGTAGGACCTTCGGTGA- 3' |
| GAPDH | Forward | 5' -GCACCGTCAAGGCTGAGAAC- 3' |
| | Reverse | 5' -TGGTGAAGACGCCAGTGGA- 3' |

**Table S8-2, Human primer sequences**

| Gene | | Primer sequence |
|---|---|---|
| p53 (exon 7-8) | Forward | 5' -ATCCTCACCATCATCACACTGG- 3' |
| | Reverse | 5' -ACAAACACGCACCTCAAAGC- 3' |
| p53 (exon 8-9) | Forward | 5' -TTTGAGGTGCGTGTTTGYGC- 3' |
| | Reverse | 5' -TTTCTTCTTTGGCTGGGGAGAG- 3' |
| p53 (exon 9-10) | Forward | 5' -CCAGCTCCTCTCCCCAGCCA- 3' |
| | Reverse | 5' -CGCTCACGCCCACGGATCTG- 3' |
| p53 (exon 11) | Forward | 5' -TTGCAATAGGTGTGCGTCAG- 3' |
| | Reverse | 5' -TCCCCACAACAAAACACCAG- 3' |
| RB1-1 | Forward | 5' -TCACATTCCTCGAAGCCCTTAC- 3' |
| | Reverse | 5' -TTTGTTGGTGTTGGCAGACC- 3' |
| RB1-2 | Forward | 5' -GCGATACAAACTTGGAGTTCGC- 3' |
| | Reverse | 5' -AAGAGCGCACGCCAATAAAG- 3' |
| RB1-3 | Forward | 5' -ATGCAGAGACACAAGCAACC- 3' |
| | Reverse | 5' -CAGACAGAAGGCGTTCACAAAG- 3' |

**Table S9, Mouse primer sequences**

| Gene | | Primer sequence |
|---|---|---|
| GADD45A | Forward | 5' -CGCTGATGCAAGGATTACAG- 3' |
| | Reverse | 5' -TTCTCGCAGCTTCCTTCTTC- 3' |
| StAR | Forward | 5' -TTGGGCATACTCAACAACCA- 3' |
| | Reverse | 5' -TGATGACCGTGTCTTTTCCA- 3' |
| CYP11A1 | Forward | 5' -GCTGGAAGGTGTAGCTCAGG- 3' |
| | Reverse | 5' -CACTGGTGTGGAACATCTGG- 3' |
| CYP11B1 | Forward | 5' -GCTTCACCATGTGCTGAAATCC- 3' |
| | Reverse | 5' -AGAAGAGAGGGCAATGTGTCA- 3' |
| GAPDH | Forward | 5' -CGGCCGCATCTTCTTGTG- 3' |
| | Reverse | 5' -GTGACCAGGCGCCCAATA- 3' |

**Supplementary Figure S1.**

The expression of steroidogenesis-related genes induced by the indicated concentration of EP in H295R cells.

H295R cells were treated with different concentration of etoposide (EP) for 3 days, followed by 1 day culturing with normal growth medium.

Relative mRNA expression of the indicated genes was analyzed by qRT-PCR.

Data are presented as the mean ± SE of 3 independent experiments.

*$P < 0.05$ vs. control.

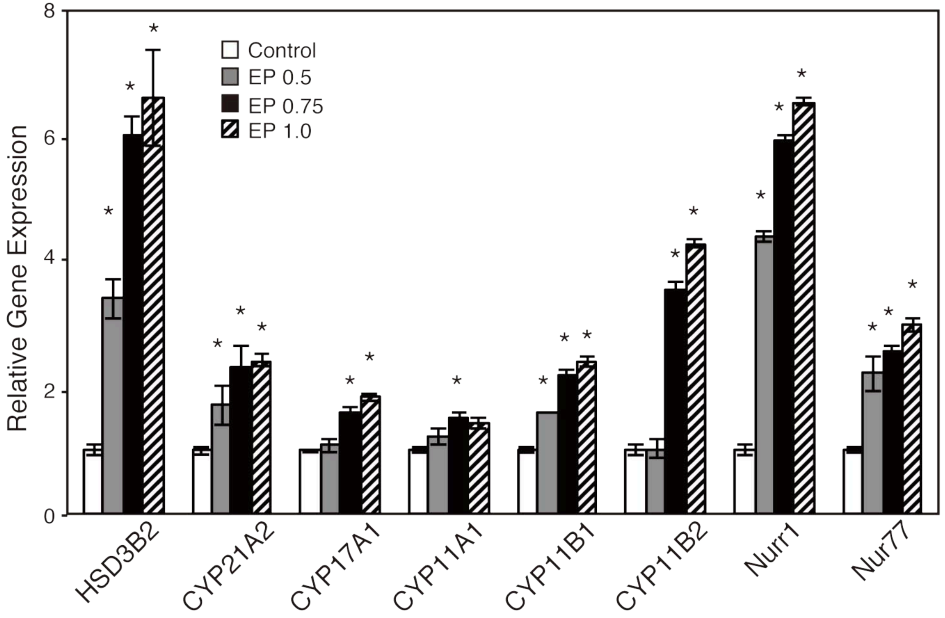

**Supplementary Figure S2.**

SF1 and StAR mRNA expression in H295R cells treated with EP for 72 h, followed by 1 day culturing, or 8-Br-cAMP for 24 h.

Relative mRNA expression of the indicated genes was analyzed by qRT-PCR. Data are presented as the mean ± SE of 3 independent experiments.

*P < 0.05 vs. control.

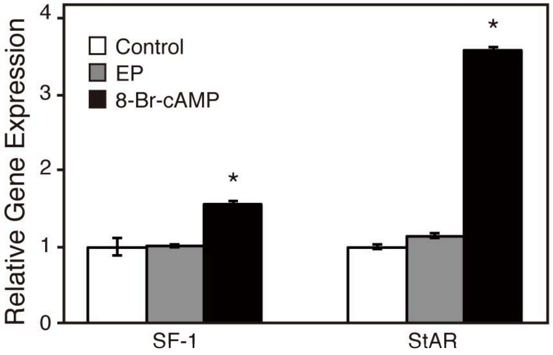

## Supplementary Figure S3

Inhibitory effect of GADD45A siRNA-2 on steroidogenesis promoted by EP H295R cells were transfected with control siRNA and siGADD45A-2, and treated with EP similarly to Figure 2.

(A) Cortisol concentration in the medium was measured using ELISA, and corrected by the number of cells. Data are presented as the mean ± SE of 3 independent experiments. *$P < 0.05$ vs. control, †$P < 0.05$ vs. EP.

(B) Relative mRNA expression of the indicated genes was analyzed by qRT-PCR. Data are presented as the mean ± SE of 3 independent experiments. *$P < 0.05$ vs. control, †$P < 0.05$ vs. EP.

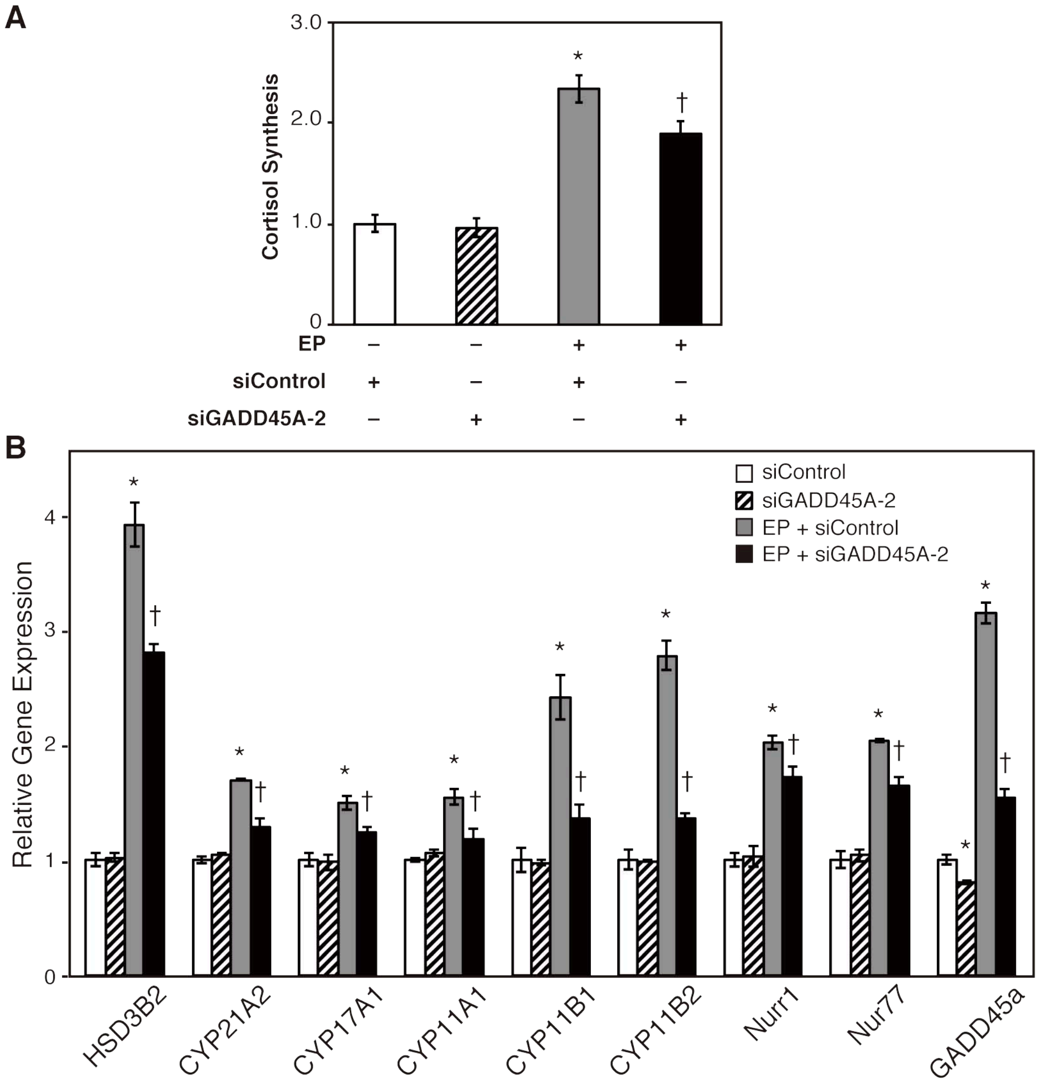

## Supplementary Figure S4

The effects of H89 on steroidogenesis promoted by 8-Br-cAMP. H295R cells were treated with 8-Br-cAMP (500 μM) and H89 (10 μM) for 24 h. Relative mRNA expression of the indicated genes was analyzed by qRT-PCR. Data are presented as the mean ± SE of 3 independent experiments. *P < 0.05 vs. control, †P < 0.05 vs. EP.

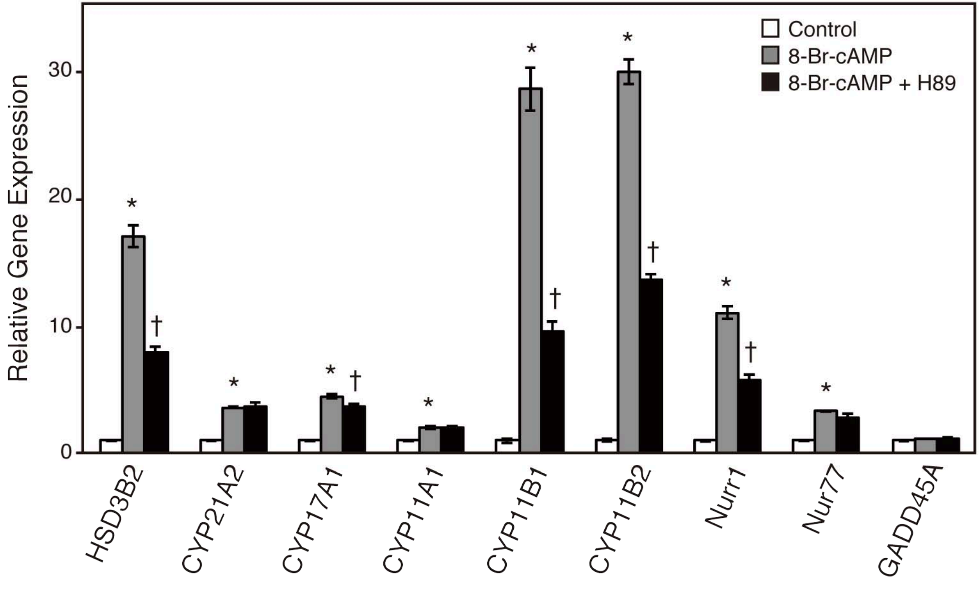

**Supplementary Figure S5**

No effect of GADD45A siRNA on steroidogenesis promoted by 8-Br-cAMP.

H295R cells were transfected with the indicated siRNAs.

After 24 h, the cells were treated with 8-Br-cAMP (500 μM) for 24 h.

Relative mRNA expression of the indicated genes was analyzed by qRT-PCR.

Data are presented as the mean ± SE of 3 independent experiments.

*P < 0.05 vs. control, †P < 0.05 vs. EP.

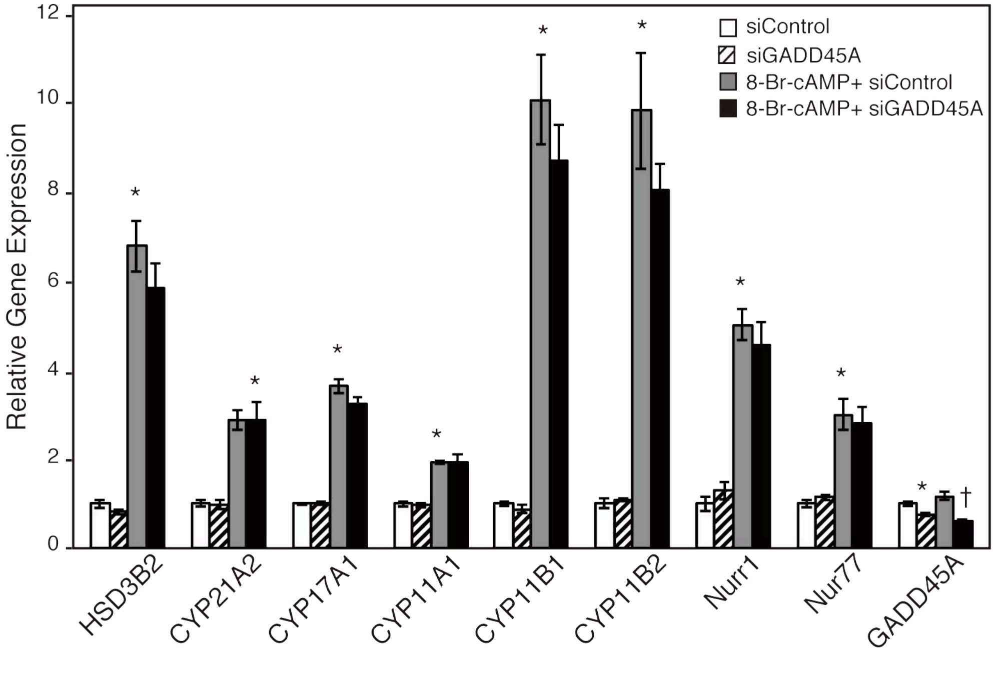

**Supplementary Figure S6**

Induction of steroidogenesis in Y1 mouse adrenocortical tumor cells.

(A) Progesterone concentration in the medium was measured using LC-MS/MS, and corrected by the number of cells. Data are presented as the mean ± SE of 3 independent experiments. *P < 0.05 vs. control.

(B) Relative mRNA expression of the indicated genes analyzed by RT-qPCR. The primer sets are described in Supplementary Table S9. Data are presented as the mean ± SE of 3 independent experiments. *P < 0.05 vs. control.

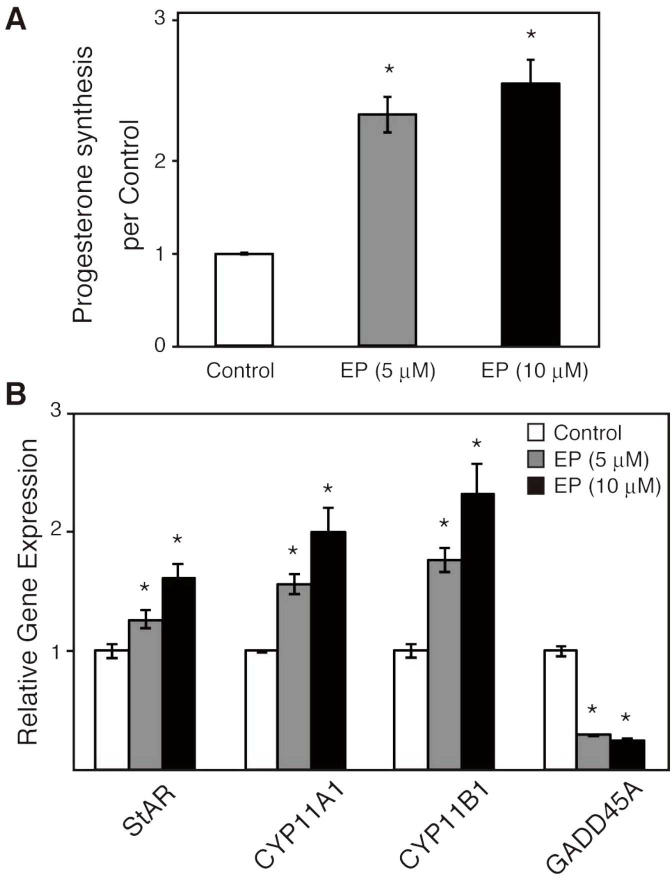

## Supplementary Figure S7

The epression of IL8, MMP10, and p16 analyzed by qRT-PCR (A), and flow cytometric analysis of the DNA content (B) in H295R cells. Cells were treated with EP (0.75) for 72 hours ( at 72h), followed by culturing without EP for 1 day (at 96h after treatment), 4 days (at 7 days after treatment), and 17 days (at 20 days after treatment).

Data are presented as the mean ± SE of 3 independent experiments.

*P < 0.05 vs. control.

**A**

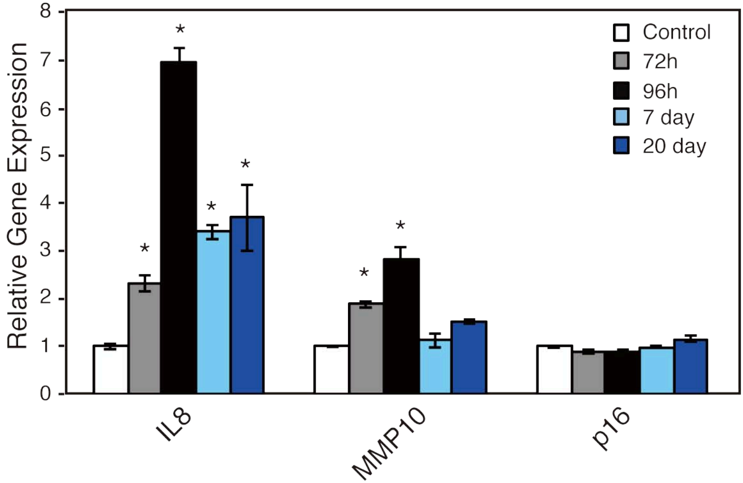


**B**

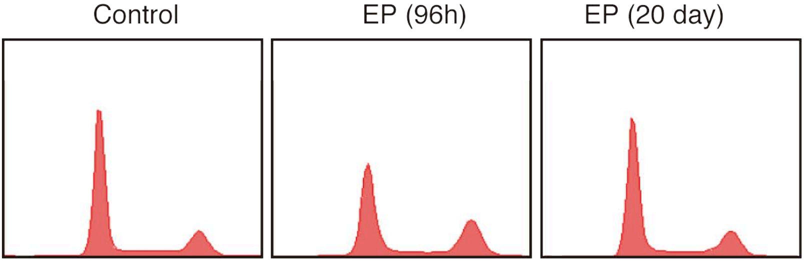


| | Control | EP (96h) | EP (20 day) |
|---|---|---|---|
| G1 (%) | 69.19 ± 0.76 | 54.78 ± 0.40* | 67.45 ± 0.24 |
| S (%) | 10.17 ± 0.26 | 11.06 ± 0.13 | 11.70 ± 0.35 |
| G2/M (%) | 20.64 ± 0.54 | 34.16 ± 0.31* | 20.85 ± 0.15 |

**Supplementary Figure S8**

**H295R cells lack p53 (exon 8-9) and RB1.**

(A) Relative p53 mRNA expression from H295R culturing in normal growth medium was analyzed by RT-qPCR using indicated primers.

(B) Relative RB1 mRNA expression was analyzed by RT-qPCR using 3 different pairs of primers.

LNCaP cells were used as the positive control, because the cells have WT of p53 and RB (Chappel et al., 2012) (Sharma et al., 2007).

Data are presented as the mean ± SE of 3 independent experiments. *P < 0.05 vs. control. ND, not detectable.

A

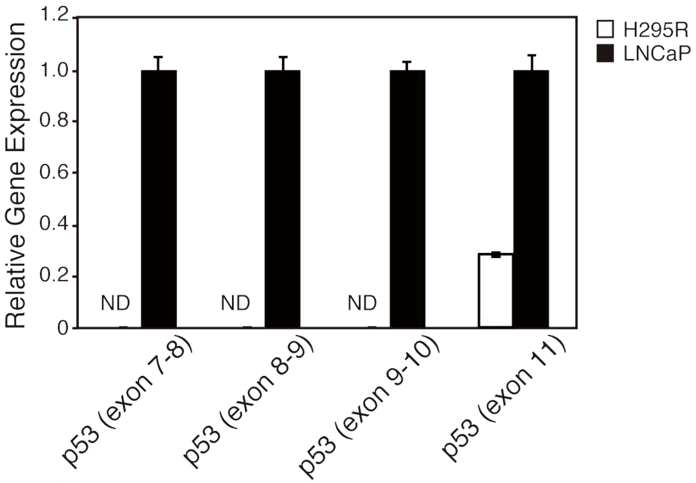


B

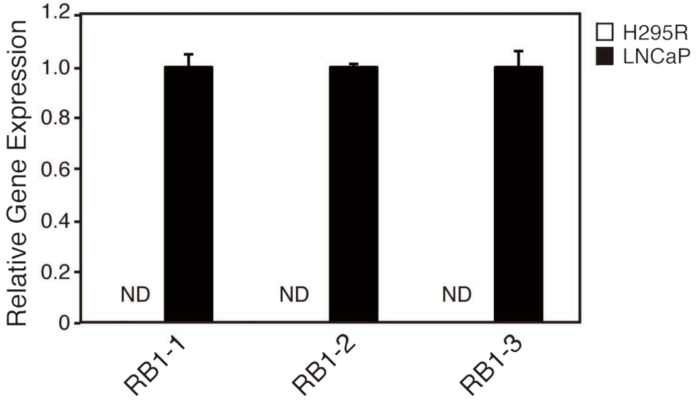

**Supplementary Figure S9**

Uncropped blots used in Figure 4A

Short exposure (A) and long exposure (B) of the same immunoblots

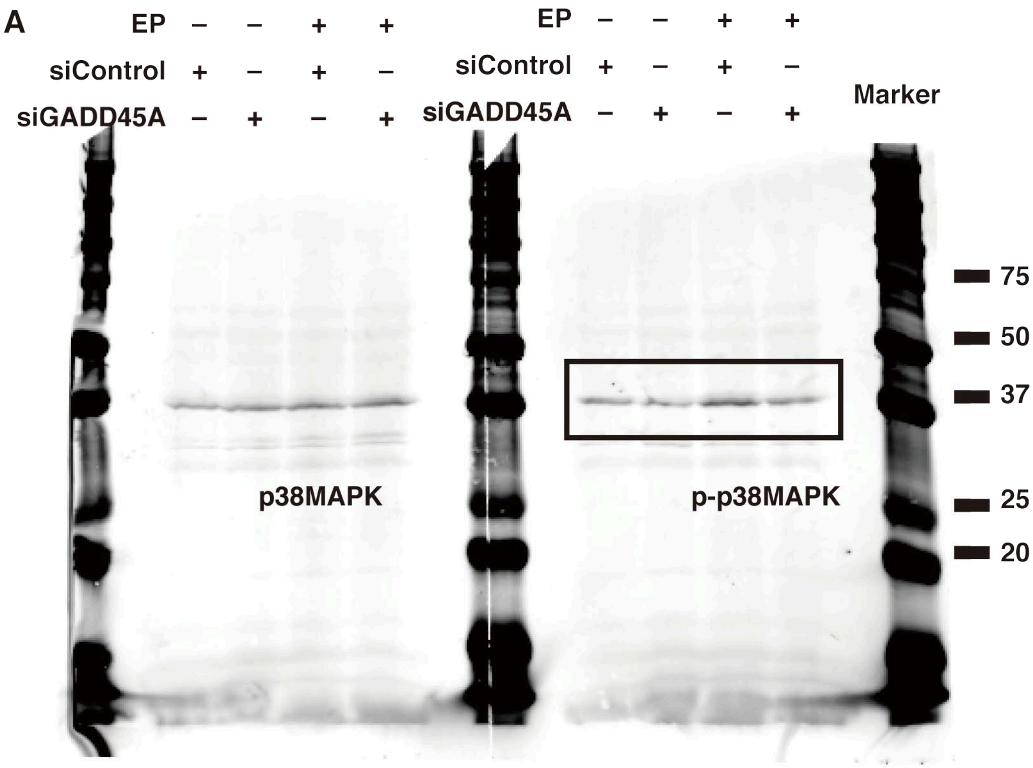


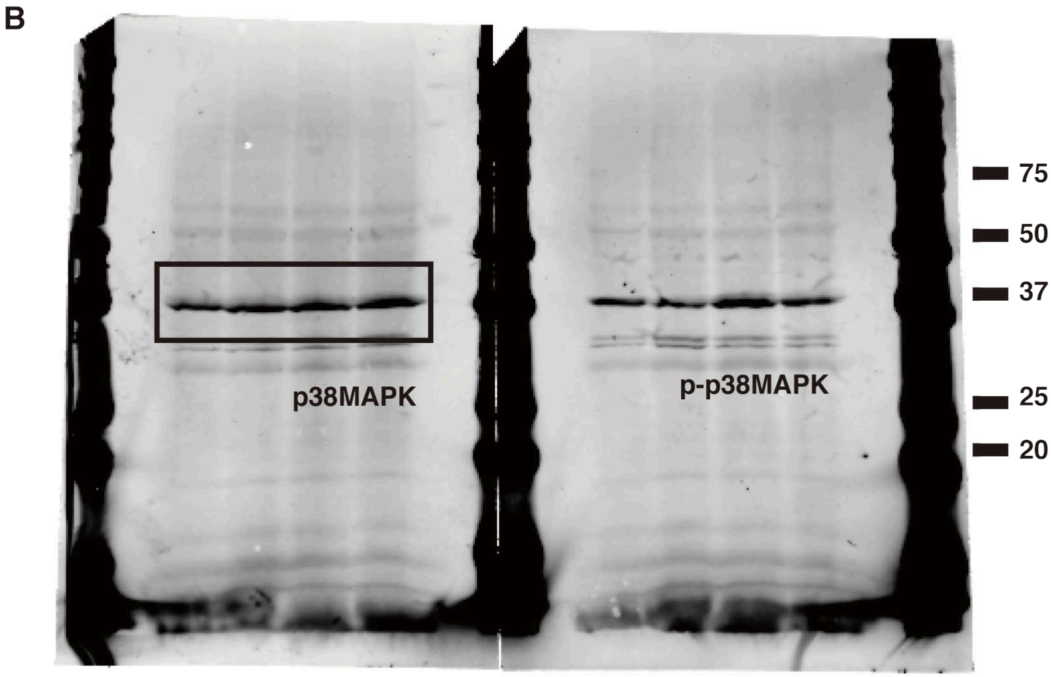

Supplement: Supplementary file 1 — Supplementary Information [file 41598_2018_27938_MOESM1_ESM.pdf]
